# Kimboza, a Small Lowland Forest With an Outstanding Herpetofauna Diversity in East Africa

**DOI:** 10.1002/ece3.70406

**Published:** 2024-10-11

**Authors:** John V. Lyakurwa, Simon P. Loader, Wilirk Ngalason, Rikki Gumbs, Caleb Ofori‐Boateng, H. Christoph Liedtke

**Affiliations:** ^1^ Department of Zoology and Wildlife Conservation University of Dar Es Salaam Dar es Salaam Tanzania; ^2^ EDGE of Existence Programme Zoological Society of London London UK; ^3^ Natural History Museum London UK; ^4^ Institute of Zoology Zoological Society of London London UK; ^5^ CSIR‐Forestry Research Institute of Ghana Kumasi Ghana; ^6^ Ecology Evolution and Development Group, Estación Biológica de Doñana (CSIC) Sevilla Spain

**Keywords:** Biogeography, Coastal forests, Eastern Arc Mountains, Herpetofauna diversity

## Abstract

The Eastern Arc Mountains (EAM) and Coastal forests of Tanzania are renowned for harboring large number of threatened and endemic vertebrate species, yet most of these areas have been partially studied. The Kimboza Nature Forest Reserve (KNFR) is a small forest which is in transition between the EAM and Coastal forests, and among the poorly surveyed areas for amphibians and reptiles. We conducted systematic surveys across the KNFR in 2012 and between 2020 and 2023 using a range of approaches with the aim of establishing a comprehensive and updated list of reptile and amphibian species and assess the contribution of EAM and Coastal forests to the KNFR's herpetofauna. We identified 77 species, 29 amphibians and 48 reptiles, substantially updating previous species lists. Three of these species (*Kinyongia magomberae*, *Trachylepis boulengeri* and *Philothamnus macrops*) represent range extensions from previously known ranges. Fourteen species are endemic to East Africa, 11 of them being restricted to Tanzania. These results make the KNFR the richest forest reserve for herpetofauna per square km in Tanzania, and most similar in its composition to the Coastal, rather than Eastern Arc forests. With the caveats concerning taxonomic uncertainties and the inequalities of sampling intensity across the region, this study shows that the KNFR is an important area that deserves conservation attention. The KNFR, like other Coastal forests, is under significant pressure from anthropogenic activities which call for an urgent action to protect this small but rich forest.

## Graphical abstract

We provide a curated checklist of amphibians and reptiles in Kimboza Nature Forest Reserve (KNFR) in Tanzania based on the new intensive fieldwork and old survey information. The KNFR boasts the richest diversity per km^2^ in Tanzania and potentially the continent. We demonstrated much of the fauna is of coastal origin but this unique fauna is highly threatened by habitat degradation.

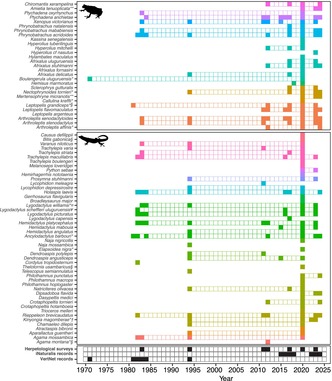

## 1 Introduction

**FIGURE 1:**
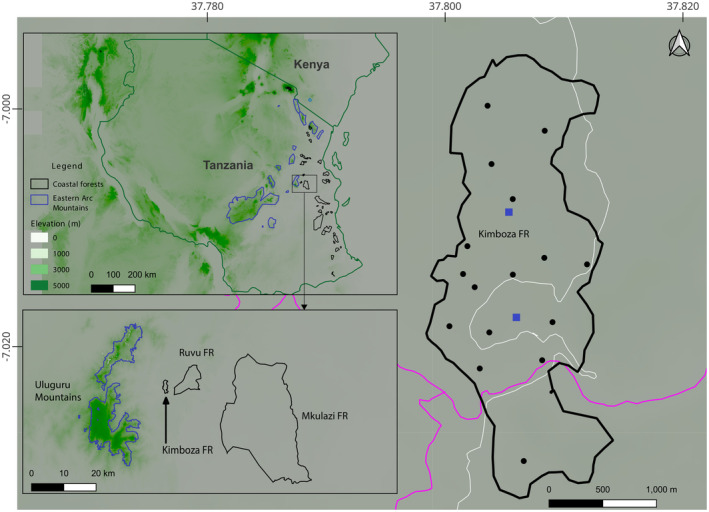
Location of the Kimboza Nature Forest Reserve and sampling sites. The blue squares indicate the sites in which bucket pitfall traps were established, and black circles are sites where time constrained searching was conducted. White line represents the Morogoro‐Kisaki road and sky blue indicates Ruvu river. Inset maps indicate position of Kimboza relative to the Eastern Arc Mountain blocks and Coastal forests of Tanzania.

Moist forests of Tanzania can be broadly grouped into at least five categories, differentiated mostly by climatic influence and edaphic factors (Lovett 1990; Menegon, Lyakurwa, and Loader 2020). Among these categories, Eastern Arc Mountains and Coastal forests are renowned for their richness in biodiversity, notably their high concentration of endemic species of plants and animals (Burgess, Clarke, and Rodgers 1998; Burgess, Fjeldså, and Botterweg 1998; Myers et al. 2000; Azeria et al. 2007; Hall et al. 2009; Gereau et al. 2016). Eastern Arc Mountains and Coastal forests can also be distinguished based on altitude (mountains vs. lowlands) and are generally geographically separated, but there are areas of continuum between these forest types (Figure 1; Lovett 1990; Clarke and Dickinson 1995; Svendsen and Hansen 1995). Forests at the base of the Eastern Arc Mountains (e.g., in the Udzungwa, Uluguru and Usambara mountain blocks) are composed of Coastal forest species and with increasing altitude gradually change to Eastern Arc Mountain species. Changes in species compositions between these two forest categories (Eastern Arc and Coastal) occur in transition zones which vary with latitude (Poynton and Boycott 1996). Some forests in Tanzania show a continuum from Coastal to Eastern Arc Mountains forest, effectively representing two biogeographical regions in one (Lovett 1990; Clarke and Dickinson 1995; Svendsen and Hansen 1995; Burgess et al. 2007; Lovett and Wasser 2008).

**FIGURE 2:**
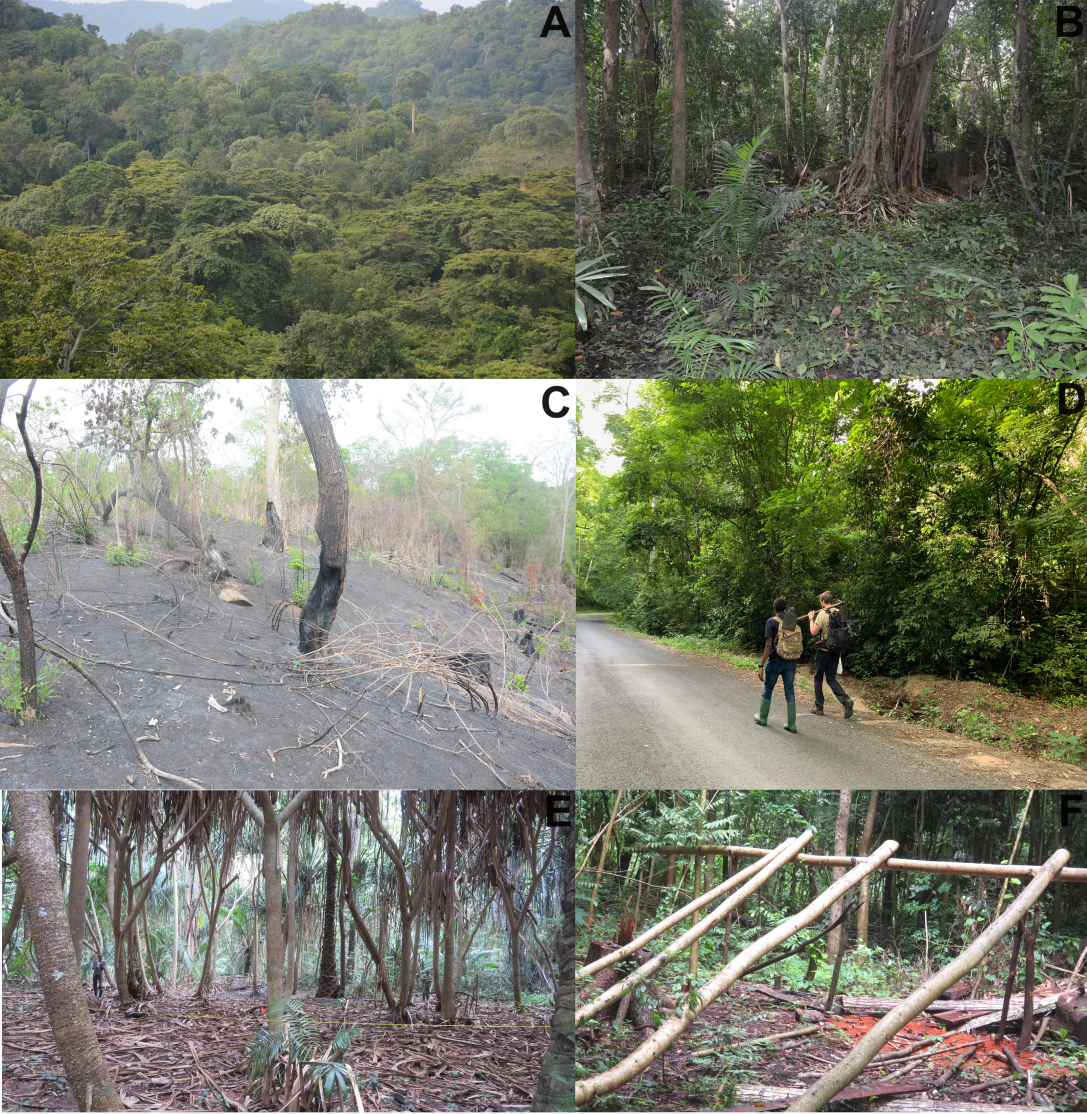
Images of the forest and human activities in the Kimboza Nature Forest Reserve. The forest as seen from above (A); one of the habitats sampled during this study (B), note the swampy foreground and the tall trees on the background; Fire in the northern part of the Kimboza Nature Forest Reserve (C); The Morogoro‐Kisaki road within the Kimboza Nature Forest Reserve (D), Note the two authors (JVL and HCL heading to a sampling site); A patch dominated by *Pandanus rabaiensis* (E), an important habitat for the endemic *Lygodactylus williamsi*; Logging within the Kimboza Nature Forest Reserve (F).

Located on the foothills of the Uluguru Mountain block, in the Central Eastern Arc Mountains, the Kimboza Nature Forest Reserve (KNFR) is among the smallest forest reserves in Tanzania (Rodgers et al. 1983; Bayliss 1994; Svendsen and Hansen 1995; Burgess and Clarke 2000; URT 2004; Rovero et al. 2014). It covers 4 km^2^ with elevation ranging from 170 to 480 m (Rodgers et al. 1983; URT 2004; Kacholi 2020; Kilawe, Mchelu, and Emily 2022) and characterized predominantly by tall Coastal forest vegetation (Rodgers et al. 1983; Werema, Howell, and Ndangalasi 2016). The origins of the fauna and flora of the KNFR are considered to be of both Coastal and Eastern Arc Mountain forests (Clarke and Dickinson 1995) given its location and history of adjoining an Eastern Arc Mountain forest—the Uluguru Mountains (Figure 1; Rodgers et al. 1983; Bayliss 1994; Clarke and Dickinson 1995; Werema, Howell, and Ndangalasi 2016). Although the KNFR is currently isolated and surrounded by heavy anthropogenic land‐use (Bayliss 1994; Werema 2014), it would have been continuous with Coastal forests and the higher elevation forests of the Uluguru Mountains prior, and this is reflected in the species found there (Rodgers et al. 1983; Bayliss 1994; Svendsen and Hansen 1995; Kacholi 2013; Werema, Howell, and Ndangalasi 2016; Kilawe et al. 2023). The KNFR has been protected as a Forest Reserve since 1964, and upgraded to a Nature Reserve just recently (URT 2024), although it has faced a number of threats across decades, with illegal harvesting (of both plants and animals), mining, road construction, bush fires and invasive plant species being the most reported ones (Figure 2; Rodgers et al. 1983; Bayliss 1994; Flecks et al. 2012; Kacholi 2020; ilawe, Mchelu, and Emily 2022).

Despite the small area of the KNFR, it is known to harbor a high number of endemic species of plants and animals (Rodgers et al. 1983; Burgess, Clarke, and Rodgers 1998; Burgess and Clarke 2000; Doggart et al. 2004) and provides a refuge for altitudinal migrant birds during cold seasons (Svendsen and Hansen 1995; Werema, Howell, and Ndangalasi 2016). Early biodiversity surveys in the KNFR mostly focused on invertebrates, plants and birds (Rodgers et al. 1983; Bayliss 1994) with plants being surveyed more than other groups. This led to recording of 17 plant species/subspecies endemic to the KNFR and 27 near endemic species (Kilawe, Mchelu, and Emily 2022) giving the area some prominence. Through these surveys, some vertebrate species were recorded by chance, including the discovery of the endemic and Critically Endangered *Lygodactylus williamsi* (Loveridge 1952). There remains a generally poor understanding of the biodiversity of this small remnant forest fragment.

Amphibians and reptiles are inadequately surveyed in the KNFR, with the total number of recorded species being relatively low compared to other similar forests (Rodgers et al. 1983; Lambert 1985; Bayliss 1994; Doggart et al. 2004; Kamungu 2018; Mkonyi 2021). The first official herpetological survey and checklist was a result of a single day visit for 3 h by Lambert (1985). This was updated 9 years later by Frontier‐Tanzania (Bayliss 1994). More recent surveys have also been published, including Kamungu (2018) and Mkonyi (2021), but their findings are fragmented, with a complete, curated amphibian and reptile species list still pending. Importantly, there are discrepancies in species identifications across the published accounts, often listing species which are not expected to be found in the area according to current guide books and databases (e.g., Spawls et al. 2018; Channing and Rödel 2019; Uetz et al. 2023; Frost 2024). Moreover, due to geographic position and biogeographic history of the KNFR there is a potential for undocumented herpetofauna species (undescribed species or unreported for the reserve).

This study aimed at establishing a comprehensive checklist of herpetofauna of the KNFR through a long‐term survey using several standardized methods, covering both wet and dry seasons, and intensive literature and database searches to collate previous published and unpublished records. By compiling these authoritative lists of amphibians and reptiles, we also aim to evaluate two questions: (1) How rich is the KNFR compared to other Coastal and Eastern Arc forests? and (2) What is the relative contribution of Eastern Arc and Coastal forest species to the KNFR fauna and speculate on the biogeographic history of the forest.

## 2 Materials and Methods

This study combines two short herpetofauna surveys (two nights, December 2012 and three nights, January 2023) with an intensive fieldwork of 11 months (December 2020 to June 2022) covering both wet and dry seasons across the whole forest in the KNFR (Figure 1). The 2012 and 2023 surveys used time constrained searching only, and the 2020–2022 involved time constrained searching in all months, and two lines of bucket pitfall trapping with a drift fence in May 2021 (see Table 1 for details). Sampling site selection ensured coverage of different microhabitat types (Figure 2; Heyer et al. 1994) and representation in terms of forest area (Figure 1). The bucket pitfall traps were set in a straight line with each line containing 11, 20 L buckets set at 5 m apart, and a transparent drift fence bisecting each bucket (*sensu* Lyakurwa et al. 2019). Time constrained searching was conducted at each site during the day and night following Lyakurwa et al. (2019) and Summay et al. (2019) (see Table 1 for details and survey efforts). Species identifications were made based on field guides (Spawls et al. 2018 for reptiles, and Channing and Rödel 2019 for amphibians), type descriptions and with the aid of molecular barcodes for 12 records. For species that were barcoded, total DNA was extracted from liver or thigh muscle tissue that was preserved in 99% ethanol using the DNeasy blood and tissue kit (Qiagen, Valencia, CA). Amplification and sequencing followed standard protocols for amphibians (e.g., Lawson 2010) and reptiles (e.g., Menegon et al. 2022). For amphibians, 16S rRNA gene was used, and for reptiles either 16S rRNA, Cytb or ND2 gene (see Table S1).

**TABLE 1:** Field surveys conducted in the Kimboza Nature Forest Reserve with amphibian/reptile records. See Table S1 for species details.

Survey	1983	1994	2011	2012	2017	2020	2023
**Surveyors**	Lambert	Frontier	Mkonyi	Loader, Menegon and Gvozdik	Kamungu and Mbije	Lyakurwa	Liedtke, Loader, Lyakurwa
**Start date/Duration (Days)**	30. Oct 1983 (1 day)	Between 28 Jan—24 March 1994	Between August and October 2011	13 and 14 Dec 2012	Between March and April 2017	11 months. Between Dec 2020 to June 2022. Dec 2020, March 2021, April 2021, May 2021, June 2021, Sept 2021, Oct 2021, Dec 2021, March 2022, April 2022, June 2022.	Jan 29–312,023 (3 days)
**Method and effort used**	Visual encounter survey ~211 min (~ 17.58 person hours)	Visual encounter surveys and Bucket pitfall traps (2 lines of 5 buckets at 2.5 m apart)	Visual encounter surveys (in 3 quadrants), acoustic surveys and opportunistic encounter	Visual encounter surveys	8 transects and 50 plots	Buckets pitfalls (660 bucket pitfall trap nights), time constrained (256 person hours, 176 night and 80 day) and opportunistic.	Time constained and opportunistic. 33 person hours of searching
**Species recorded**	13	25	10	15	13	69	27
**Specimens collected**	6	60	na	65	93	25	6
**Specimens housed at**	Natural History Museum, London	Natural History Museum, London and University of Dar es Salaam	University of Dar es Salaam	University of Dar es Salaam	Sokoine University of Agriculture Zoology	University of Dar es Salaam	University of Dar es Salaam
**Resulting publications**	Lambert 1985	none	Mkonyi 2021	none	Mbije 2021; Mbije and Kamungu 2021		
**Notes**	Only a day seach, mostly close to the main road.	A part of general biodiversity surveys covering range of taxa. Special focus on mammals, birds, amphibians, reptiles and invertebrates. Behavioral study for *Lygodactylus williamsi* was also covered. Reported a number of species not expected to be found there by the current range maps. E.g *Cryptoblepharu*s, *Leptotyphlops longicaudatus*, *Agama agama ufipae*, *Holodactylus africanus* and *Duberria lutrix shirana.*	A study to update amphibian and reptile biodiversity in 17 forests of the Uluguru Mountains. Only during the dry season. Recorded the only *Lycophidion meleagre*. No record of *Lygodactylus williamsi* which is iconic and failrly common in the reserve.	A part of general biodiversity surveys covering range of amphibians and reptiles.	MSc study by Kamungu focused on understanding distribution and feeding ecology of Anurans in Kimboza. Some few missidentifications, e.g., Leptopelis uluguruensis instead of *Leptopelis grandiceps*, *Arthroleptis affinis* instead of *A. stenodactylus* and *Phrynobatrachus acridoides* instead *P. mababiensis.*	Started as a two year fellowship to study the ecology of *Lygodactylus williamsi* but later expanded to document the overall herpetofauna diversity in the forest.	A part of general biodiversity surveys covering range of amphibians and reptiles.

*Note:* “none” indicates no publication has been made and “na” indicates uncertainty on how many specimens were collected.

We also compiled information on other surveys conducted in the KNFR from the literature and biodiversity databases. We queried biodiversity databases (GBIF; www.gbif.org (https://doi.org/10.15468/dl.t26vw9, and https://doi.org/10.15468/dl.kth9yd), VertNet; www.vertnet.org, iNaturalist; www.inaturalist.org) and Google Scholar between March 10 and May 18 2024 using a combination of “Amphibia/Reptilia/Herpetofauna” and the reserve's boundary to find any record of these taxa from the forest reserve. We also queried the biodiversity database of the University of Dar es Salaam, and museum records of the Natural History Museum in London to access records of specimens deposited by previous researchers (e.g., Rodgers et al. 1983; Bayliss 1994; Doggart et al. 2004). All resulting species lists were curated to use the current nomenclature (Frost 2024 for amphibians and Uetz et al. 2023 for reptiles) and resolve species with uncertain identities, e.g., those which were not identified to species level, undergone taxonomic revisions or represent mis‐identifications. This involved examining preserved materials and photographs, contacting species experts, consulting the Amphibian Species of the World Website (Frost 2024) and the Reptile Database (Uetz et al. 2023) and performing genetic barcoding where possible (see Table S1). These led to exclusion of some species with records from the KNFR as they proved to be mis‐identifications or not supported by the current sources, e.g., *Arthroleptis sylvatica* (now *Arthroleptis sylvaticus*), *Leptopelis parkeri*, *Phrynobatrachus parvulus*, *Hyperolius puncticulatus*, *Agama agama ufipae* (now *Agama dodomae*), *Holodactylus africanus*, *Cryptoblepharus* sp., *Leptotyphlops longicaudus* (now *Myriopholis longicauda*) and *Duberria lutrix*. Some of these species are not considered to be found in East Africa according to the current distribution maps, e.g., *Arthroleptis sylvaticus* (Channing and Rödel 2019; Frost 2024) and *Myriopholis longicauda* (Spawls et al. 2018; Uetz et al. 2023). *Arthroleptis sylvaticus* was considered a synonym of *A. xenodactylus* by Loveridge (1957), a widely distributed species in Tanzania, but currently *A. sylvaticus* and *A. xenodactylus* are recognized as separate species with the former being restricted to central and west Africa, and the latter to the Eastern Arc Mountains of Tanzania (Frost 2024). Other species are found in Tanzania but restricted to other regions far from the KNFR, e.g., *Agama agama ufipae* (now *Agama dodomae*), *Duberria lutrix* and *Holodactylus africanus* (Spawls et al. 2018; Uetz et al. 2023). Records of *Holaspis guentheri* from the VertNet database were updated to *Holaspis laevis*; the latter was a subspecies of the former but now two different species, the former being known from a single locality in Tanzania, close to the Tanzania‐Uganda border and the latter being common along the coast (Spawls et al. 2018). The only known *Cryptoblepharus* in Tanzania (*Cryptoblepharus africanus*) is limited to the coral rag and does not occur beyond the intertidal zone of the Indian ocean, and its inclusion in the KNFR in the past might be a mis‐identification. *Leptopelis uluguruensis* reported by Kamungu (2018), Mbije (2021), Mbije and Kamungu (2021) is a misidentified *L. grandiceps* (see Page 35 plate 1 L in Kamungu 2018). The same authors misidentified *Arthroleptis stenodactylus* as *A. affinis* (page 34 plate 1d in Kamungu 2018) and *Phrynobatrachus mababiensis* as *P. acridoides* (page 35 plate 1 m in Kamungu 2018). Existing *Xenopus* records in the KNFR previously represented *X. borealis*, *X. laevis* and *X. muelleri* but after photos were reviewed by Ben Evans (pers. comm.), an expert on this group, all records seem to represent only one species, and are assigned to *X. victorianus*. By combining the current surveys and all available records, we compile a definitive species list, indicating the IUCN status and endemism for all species and provide notes where more future research is needed (see Table S1).

To show the adequacy of sampling and species records over the years 1983–2023, a species accumulation curve was generated. To understand the position of the KNFR in terms of herpetofauna species when compared to other Coastal and Eastern Arc forests, we compare its species richness to records of amphibians and reptiles from Coastal forests of Tanzania and Eastern Arc Mountain blocks from Burgess and Clarke (2000) and Rovero et al. (2014) (see Table S2). We supplemented these two sources by searching for other published and unpublished reports in Google scholar, and occurrence data from VertNet and GBIF between 10 March to 18 May 2024 using the name of each forest (see Burgess and Clarke (2000) and Rovero et al. (2014) for the list of forests used) together with the word amphibia/reptilia. This was important since there has been an extensive accumulation of records from these forests since 2000/2014 and also because Rovero et al. (2014) only listed endemic/near endemic species (see Table S2). We compared forest species richness with forest area (in squared kilometers), with forest sizes obtained from Clarke and Dickinson (1995), Burgess and Clarke (2000) and Rovero et al. (2014). We then compared species compositions of the different forests using Nonmetric Multidimensional Scaling (NMDS) based on the Bray–Curtis dissimilarity index calculated from the species presence–absence matrix. This was performed using the metaMDS() function of the vegan package v2.6–4 (Oksanen et al. 2022) implemented in R v4.3.1.

## 3 Results

### 3.1 Previous Surveys

**FIGURE 3:**
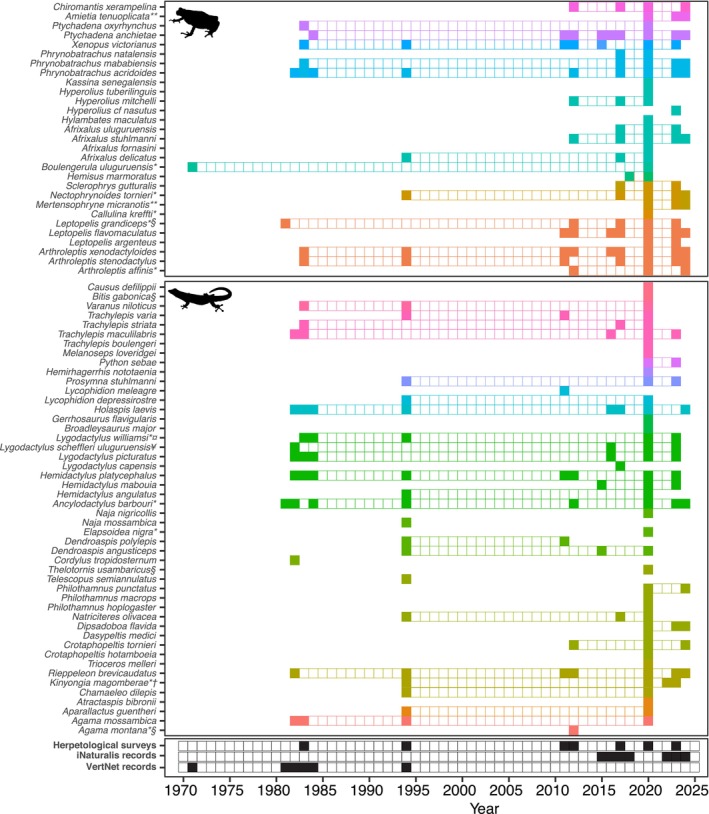
Amphibian and reptile species detection through time in the Kimboza Nature Forest Reserve. Grids span from the first to last year a species has been recorded (using the methods mentioned at the bottom panel of the figure), with filled grids representing confirmed sightings. Colors refer to taxonomic families and species are divided into amphibians (top panel) and reptiles (middle panel). The bottom panel indicates years for which intentional herpetological surveys were conducted versus additional species records derived from iNaturalist and VertNet. Species names are annotated to indicate IUCN Red List status and endemism, where § = Vulnerable, †Endangered, ¤Critically Endangered, * = Tanzania endemic, ** = East African Endemic.

Four surveys have been conducted in the KNFR apart from this study (Figure 3; Table 1 and Table S1). One survey focused only on amphibians while the rest covered both amphibians and reptiles (Table 1). Collectively, these surveys recorded 16 amphibian species from seven families and 22 reptile species from 11 families. However, among amphibians, only *Arthroleptis xenodactyloides* was recorded throughout all four surveys, with *Arthroleptis stenodactylus* and *Xenopus victorianus* missed by one survey. Four species were recorded by half of the surveys and the rest (nine species) were recorded only by a single survey (Table S1; Figure 3). For reptiles, only one species (*Hemidactylus platycephalus*) was recorded by all previous surveys, followed by seven species that were recorded by two surveys (only missed in one survey), the rest (14 species) were recorded by only one survey (Table S1).

### 3.2 Current Surveys

The current surveys have documented a total of 71 species (29 amphibians from 10 families and 42 reptiles from 14 families) (Table S1; Figure 3). Thirteen amphibian and 24 reptile species are reported for the first time in the KNFR. This also includes one previously missed order (Gymnophiona; *Boulengerula uluguruensis*), three amphibian families (Brevicipitidae, Hemisotidae and Herpelidae) and three reptilian families (Gerrhosauridae, Prosymnidae and Viperidae) (Table S1). All species recorded by the previous surveys are confirmed to occur in the reserve by the current surveys except four species (*Telescopus semiannulatus*, *Dendroaspis polylepis*, *Naja mossambica* and *Lycophidion meleagre*) (Table S1). Surprisingly, all four are snakes and were only recorded by a single survey except *Dendroaspis polylepis*, which is known from two surveys. *Lycophidion meleagre* is a poorly known and secretive snake, and the two elapids (*Dendroaspis polylepis* and *Naja mossambica*) are unlikely to thrive in such a small and isolated forest. *Naja mossambica* is also found a bit south and its record in Kimboza needs confirmation. Fifteen species (*Atractaspis bibronii*, *Bitis gabonica*, *Broadleysaurus major*, *Chamaeleo dilepis*, *Elapsoidea nigra*, *Melanoseps loveridgei*, *Philothamnus hoplogaster*, *Trioceros melleri*, *Dasypeltis medici*, *Hyperolius cf. nasutus*, *Philothamnus punctatus*, *Trachylepis boulengeri*, *Aparallactus guentheri*, *Gerrhosaurus flavigularis* and *Philothamnus macrops*), recorded by the current surveys are singletons while six are doubletons (*Agama mossambica*, *Afrixalus delicatus*, *Boulengerula uluguruensis*, *Crotaphopeltis hotamboeia*, *Mertensophryne micranotis* and *Python sebae*). The rest were represented by at least three individuals.

Three species *Kinyongia magomberae*, *Trachylepis boulengeri* and *Philothamnus macrops* represent range extensions from previously known ranges. The former was reported as *Kinyongia oxyrhina* by Bayliss (1994), but this study has confirmed through both morphological examination and DNA analysis (99.1% BLAST match for 16S rRNA) that this population belongs to *Kinyongia magomberae*, a range extension of more than 128 km from the previous range (Magombera forest and lowland Udzungwa Mountains National Park). We also report the first case of uniformly black (melanistic) *Philothamnus macrops*, and an extremely big *Philothamnus punctatus* (~ 1.5 m).

We also retrieved 79 records from the VertNet database (11 amphibians and 68 reptiles) contributed mostly by Sam Telford, M.R. Lambert, Frontier, Stolze and Scharf covering 6 years (1971, 1981–1984 and 1994) and 67 records from iNaturalist covering October 2015 to March 2024 (Table S1). These added two species (*Cordylus tropidosternum* and *Lygodactylus capensis*) that were not found during the current surveys (Table S2).

**FIGURE 4:**
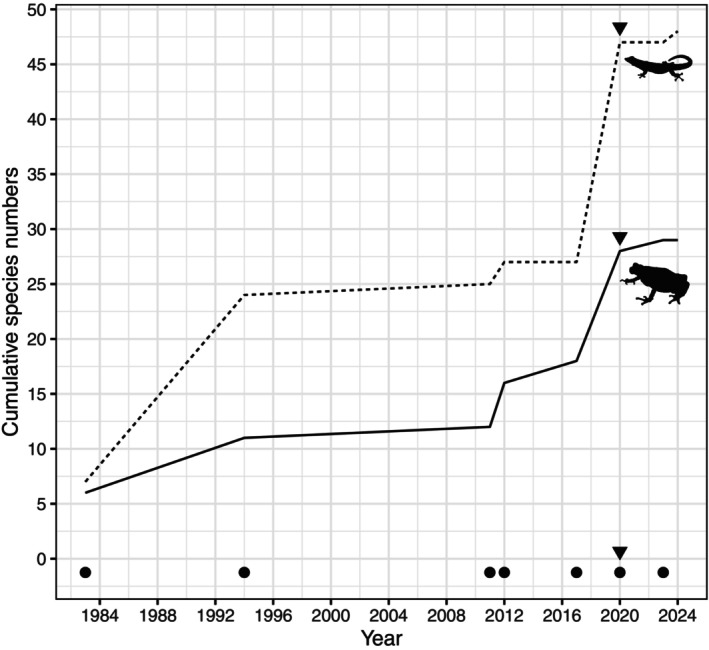
Cumulative numbers of amphibian and reptile species recorded across dedicated herpetological surveys in the Kimboza Nature Forest Reserve, for amphibians (solid line) and reptiles (dashed line). Note the dramatic increase in species in 2020 (arrow heads). The black points indicate the dates of the herpetological surveys.

We update the checklist of amphibians and reptiles for the KNFR to 77 species (29 amphibians and 48 reptiles). Hyperoliidae and Artholeptidae are the most dominant amphibian families while for reptiles, these are Colubridae and Gekkonidae (Table S1). However, species accumulation curve across surveys has not reached asymptote (Figure 4) indicating chances of adding more species to the list with more survey efforts. Fourteen of the recorded species are endemic to East Africa, 11 of them being restricted to Tanzania (Table S1). Eight species are threatened with extinction, one is Data Deficient, and the rest are listed as Least Concern by IUCN Red List (IUCN 2024). Nineteen species are forest species, while the rest are found in mixed habitats, e.g., forest and woodland, savanna and grassland. Eight species are predominantly found in the Eastern Arc Mountains, six are shared between Eastern Arc Mountains and Coastal forests, 12 are predominantly Coastal and the rest are widely distributed (Table S1).

**FIGURE 5:**
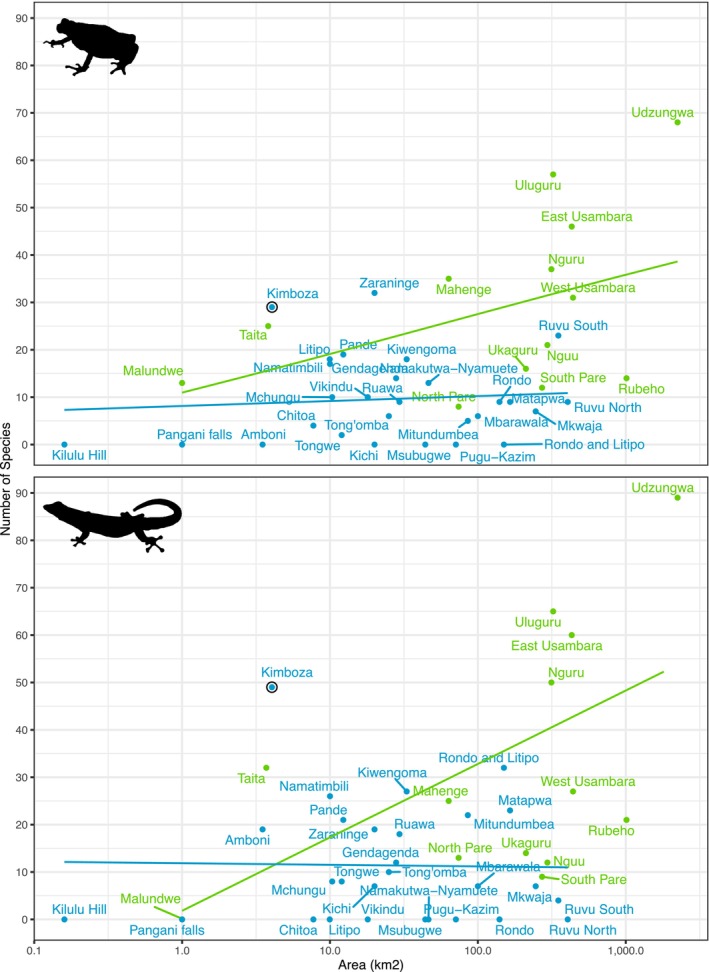
Relationship between herpetofauna species richness and forest area (in squared kilometers) for Eastern Arc Mountain blocks and Coastal forests of mainland Tanzania. Top panel shows amphibians, bottom panel shows reptiles. Green = EAM and Blue = Coastal forests. The solid lines indicate a linear regression lines and text labels indicate forest names. See Table S2 for details of each forest and data sources.

Using compiled information of amphibian and reptile distribution in the Eastern Arc Mountains and the Coastal forests of Tanzania (Table S2), we fitted linear models to test the effects of forest types (Eastern Arc Mountain vs. Coastal) and forest size (log transformed square kilometers) on species richness (Figure 5). Models were fitted independently for amphibians (*F*‐statistic: 12.97 on 2 and 31 DF, *p* < 0.001) and reptiles (*F*‐statistic: 9.034 on 2 and 27 DF, *p* < 0.001). We found that the number of species when controlling for forest size, is significantly lower in Coastal forests compared to Eastern Arc Mountain forests for amphibians (*t* = −2.387, *p* = 0.023), but not for reptiles (*t* = −1.099, *p* = 0.281). However, for both taxa there was a positive relationship between species numbers and forest area (*t* value = 3.071, *p* = 0.004; *t* value = 3.140, *p* = 0.004). It is also notable that the KNFR stands out as having substantially higher species numbers per km^2^, than other forests.

**FIGURE 6:**
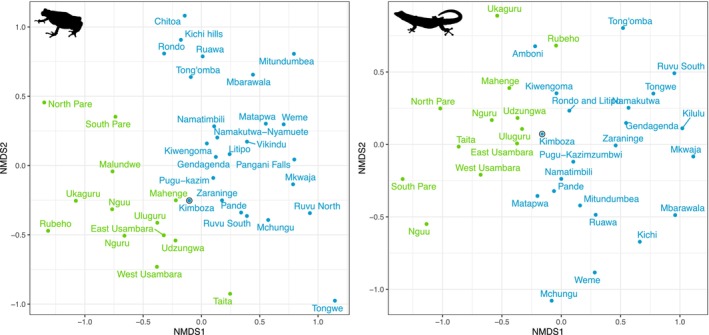
Species compositions of the different Eastern Arc Mountain blocks and Coastal forests using Nonmetric Multidimensional (NMDS) Scaling based on the Bray–Curtis dissimilarity index calculated from the species presence–absence matrix. Left panel shows amphibians, right panel shows reptiles. Forest names are provided and Kimboza is highlighted with a black outline. Colours indicate forest type, where green = EAM and blue = Coastal forest.

The Nonmetric multidimensional scaling (NMDS) based on the Bray‐Curtis distances for amphibians and reptiles show a clear partitioning of Eastern Arc and Coastal species assemblages (Figure 6). Interestingly, the KNFR shows a position close to the boundary of this division and reflects its closer affiliation to Eastern Arc Mountain forests compared to other coastal locations.

## 4 Discussion

### 4.1 General

Through dedicated herpetological surveys over a period of 3 years and an intensive literature and database search, we document a total of 77 species, 29 amphibians and 48 reptiles in one of the smallest Forest Reserves of Tanzania. This represents 22.1% of all amphibian species (of 131 species) and 43.3% of all reptile species (of 171 species) recorded in the Eastern Arc Mountains and Coastal forests of mainland Tanzania (Clarke 1995; Burgess, Clarke, and Rodgers 1998; Burgess, Fjeldså, and Botterweg 1998; Burgess and Clarke 2000; Msuya 2001, 2003; Loader, Poynton, and Mariaux 2004; Msuya et al. 2004; Doggart et al. 2006; Poynton et al. 2007; Menegon, Doggart, and Owen 2008; Loader et al. 2011; Howell et al. 2012; Gereau et al. 2016; Barratt 2017; Lyakurwa et al. 2019). More than half of these new records were singletons, having only been recorded on a single occasion. This may indicate that these species are enigmatic and may seldomly be recorded. Specialized behavior such as fossorial lifestyles or high levels of crypsis may explain this. Species like *Melanoseps loveridgei*, *Trioceros melleri*, *Aparallactus guentheri* and *Bitis gabonica* are well known for their secretive behaviors. Alternatively, it may indicate that many species, although present, occur in low numbers and are thus generally rare. Given the small size of the reserve, the threats and isolation from other nearby forests, the large number of singletons, doubletons and missing species may be at risk of ongoing or future local extinction and this needs to be monitored.

Almost all species recorded in the KNFR are also known from at least one of the coastal forests of mainland Tanzania, except records of *Afrixalus uluguruensis*, *Leptopelis grandiceps*, *Nectophrynoides tornieri*, *Agama montana*, *Kinyongia magomberae*, *Lygodactylus williamsi*, *Naja nigricollis* and *Lycophidion meleagre*. Most of these species (except *Naja nigricollis*) are usually associated with forests on the base of Eastern Arc Mountains and there are no documented records in the coastal forests of mainland Tanzania (Clarke 1995; Broadley and Howell 2000; Burgess and Clarke 2000; Poynton 2000; Msuya 2001, 2003; Msuya et al. 2004; Barratt 2017). Some of these records might in the future reflect species with more refined distributions—potentially even unique to the KNFR, given the taxonomic uncertainty of many species. For example, each of *Callulina cf. kreffti* and *Nectophrynoides tornieri* probably represents more than one species (Loader et al. 2010; Liedtke et al. 2016; Menegon, Lyakurwa, and Loader 2020).

Despite the KNFR being a small forest fragment, it consists of multiple habitat‐types that host highly specialized species (e.g., *Lygodactylus williamsi* in *Pandanus* plants (Figure 2; Bayliss 1994; Flecks et al. 2012)). It has large and continuous rock crevices and caves which limit sampling and might hide species from detection. It is not known whether previous surveys were restricted largely to the road that traverses the forest and thus may have missed important patches of this heterogeneous forest.

### 4.2 How Rich Is the KNFR Compared to Other Forest Locations in Tanzania

The high concentration of species in this forest can be better understood when number of species and forest area across EAM and Coastal forests are considered (see Table S2; Figure 5). In the KNFR, there is a remarkable 19 species per square kilometer (11.9 reptile and 7.2 amphibians per square kilometer). Most other forests are significantly larger than the KNFR with reduced concentrations of herpetofauna species, e.g., less than 2 species per square kilometer for Coastal forests (Clarke 1995; Broadley and Howell 2000; Burgess and Clarke 2000; Poynton 2000; Msuya 2001, 2003; Barratt 2017) and less than 1 species per square kilometer for Eastern Arc Mountains (Loader, Poynton, and Mariaux 2004; Menegon, Doggart, and Owen 2008; Lawson and Collett 2011; Ngalason and Mkonyi 2011; Lyakurwa et al. 2019; Tonelli 2021; Liedtke et al. 2022; Christopher, Ngalason, and Lyakurwa 2023). Based on the current estimates, the KNFR has the highest concentration of herpetofauna species per square kilometer in mainland Tanzania (highlighted in Figure 5). Given the rich biodiversity of Tanzania (Meng et al. 2016; Uetz et al. 2023; Frost 2024), on a continental scale, the KNFR is therefore a significant site for amphibian and reptile diversity in all of Africa.

Our finding that the KNFR is disproportionately rich in species comes with some caveats which might skew this result. As is the case for many estimates of tropical diversity, there are significant shortfalls in our understanding of species which might underestimate the true diversity but also the distribution of these species units (Mora et al. 2011; Carné and Vieites 2024). Wallacean and Linnean shortfalls in knowledge will likely change the findings outline here. Most likely impacts will be two aspects: (1) Better sampling across all sites (e.g., Wallacean shortfall) will elevate overall diversity of other locations reducing the significance of the KNFR and (2) Refined understanding of species units (e.g., Linnean shortfall) will demonstrate a better understanding of shared similarity across geographical locations and provide more clarity on geographic relationships (Hortal et al. 2015). Most Coastal forests are poorly surveyed, especially on herpetological context, and some groups have taxonomic problems (Poynton 2000; Azeria et al. 2007; Wegner et al. 2009; Howell et al. 2012; Gereau et al. 2016; Barratt et al. 2018).

### 4.3 Is Kimboza Nature Forest Reserve a Coastal Forest?

The spectacular diversity of the KNFR appears to be linked to its geographical position, sitting on the foothills of the Uluguru mountains and adjacent to the patchy remnant forests of the Coastal region. Our analysis of species presence and absence data allows us to better understand the relative contributions of these biogeographical regions. We show most amphibians and reptiles of the KNFR to be shared by other forests along the coast. This closer link makes logical sense given the relative conditions of the KNFR location (low elevation conditions) and species adapted to these conditions. Eastern Arc species are fewer and for the species penetrating this region are the lower altitude adapted species (lowland and submontane species), rather than the higher elevation species. This pattern is also reflected in other taxonomic groups, supporting our finding (e.g., Rodgers et al. 1983; Kacholi 2013).

Future work will need to examine the evolutionary history of species occurring across this region to look at the timing of biogeographical events between the KNFR and adjoining areas. For example, are all Eastern Arc species found in the KNFR the result of recent biogeographic events, potentially linking to recent Pleistocene fluctuations, which might have provided opportunities for higher elevation species to disperse into lower elevation forests? Or what is the possible origin of species like *Kinyongia magomberae* in Kimboza? Although *K. magomberae* is a lowland species, its patchy distribution between Kimboza and lowland Udzungwa, with no population in between, might pose several interesting phylogeographic questions. It is likely the species was widely distributed on the lowland forests since it is the only *Kinyongia* known from that elevation range in Tanzania (Spawls et al. 2018). There is a good opportunity to understand the origins of this fascinating biota through phylogeographic and population genetic approaches.

### 4.4 Conclusions

This study outlines the importance of dedicated survey efforts to understand biodiversity. Without collecting data using different methodologies, across different habitats, over seasons, many species would have remained elusive. Generally, Tanzanian Coastal forests are poorly known and have not received the type of intensive surveying outlined in this study and is required to better understand biodiversity and its change in the face of rapid environmental change (e.g., Ruvu South (Barratt et al. 2017)).

For many years, *Lygodactylus williamsi* has been used as an icon to highlight the conservation importance of the KNFR. This gecko is among the most threatened reptiles in Africa, especially due to illegal trade and habitat loss (Flecks et al. 2012; Auliya et al. 2016; Meiri et al. 2017) and the KNFR has been the most important site for its conservation. This study brings more attention to this forest, because it now reveals not only high concentration of species but also the presence of other endemic species (14 East African endemics, 11 of them restricted to Tanzania).

The KNFR represents a significant area for amphibian and reptile diversity. More broadly, the KNFR is also important for other taxonomic groups and is an important repository for biodiversity. The Forest Reserve is currently isolated from other forests (both the Eastern Arc and Coastal forests), meaning future existence of these species will depend on the proper protection strategies for this “island of diversity”. Kimboza is currently facing some significant conservation issues including fire, invasive plant species, deforestation, species exploitation, and mining (Kilawe et al. 2023). More determined efforts are needed to combat fire occurrences and control the invasive *Cedrela odorata* (see Werema 2014; Kilawe, Mchelu, and Emily 2022; Kilawe et al. 2023). The forest is also accessible with a major road bisecting it, providing easy access to the forest and potentially promoting fragmentation of an already a very small forest. It requires a determined effort to monitor and protect this habitat to ensure its long‐term survival.

## Author Contributions

**John V. Lyakurwa:** conceptualization (equal), data curation (equal), formal analysis (equal), funding acquisition (equal), investigation (equal), methodology (equal), writing – original draft (equal), writing – review and editing (equal). **Simon P. Loader:** conceptualization (equal), investigation (equal), methodology (equal), writing – original draft (equal), writing – review and editing (equal). **Wilirk Ngalason:** conceptualization (equal), methodology (equal), project administration (equal), writing – review and editing (equal). **Rikki Gumbs:** conceptualization (equal), funding acquisition (equal), project administration (equal), writing – review and editing (equal). **Caleb Ofori‐Boateng:** conceptualization (equal), funding acquisition (equal), project administration (equal), writing – review and editing (equal). **H. Christoph Liedtke:** conceptualization (equal), data curation (equal), formal analysis (equal), investigation (equal), methodology (equal), visualization (equal), writing – original draft (equal), writing – review and editing (equal).

## Conflicts of Interest

The authors declare no conflicts of interest.

## Supporting information


**Table S1.** Checklist of amphibians and reptiles of Kimboza Forest Reserve. Note: EAM = Eastern Arc Mountains * = Tanzanian Endemic, ** = East African Endemic, § = VU, † = EN, ¤ = CR, ¥ = DD.

**Table S2.** Forest area, bibliography and checklist of amphibians and reptiles of Eastern Arc Mountains and Coastal forests of Mainland Tanzania.

## Data Availability

Molecular sequences have been deposited on GenBank (accession numbers: PQ280309‐PQ280319). All other data related to the manuscript is included as supplementary tables and can be accessed via links on ‘supporting information’ section.
